# Novel genetic reassortants in H9N2 influenza A viruses and their diverse pathogenicity to mice

**DOI:** 10.1186/1743-422X-8-505

**Published:** 2011-11-04

**Authors:** Yuhai Bi, Lu Lu, Jing Li, Yanbo Yin, Yi Zhang, Huijie Gao, Zhuoming Qin, Basit Zeshan, Jinhua Liu, Lei Sun, Wenjun Liu

**Affiliations:** 1 Center for Molecular Virology, Key Laboratory of Pathogenic Microbiology and Immunology, Institute of Microbiology, Chinese Academy of Sciences, Beijing 100101, China; 2 Graduate University of Chinese Academy of Sciences, Beijing 100101, China; 3 College of Animal Science and Veterinary Medicine, Qingdao Agricultural University, Qingdao 266109, China; 4 Institute of Animal Science and Veterinary Medicine, Shandong Academy of Agricultural Sciences, Jinan, Shandong 250100, China; 5 Key Laboratory of Zoonosis of Ministry of Agriculture, College of Veterinary Medicine, China Agricultural University, Beijing 100193, China

**Keywords:** avian influenza virus, H9N2, reassortant, genotype, pathogenicity

## Abstract

**Background:**

H9N2 influenza A viruses have undergone extensive reassortments in different host species, and could lead to the epidemics or pandemics with the potential emergence of novel viruses.

**Methods:**

To understand the genetic and pathogenic features of early and current circulating H9N2 viruses, 15 representative H9N2 viruses isolated from diseased chickens in northern China between 1998 and 2010 were characterized and compared with all Chinese H9N2 viruses available in the NCBI database. Then, the representative viruses of different genotypes were selected to study the pathogenicity in mice with the aim to investigate the adaptation and the potential pathogenicity of the novel H9N2 reassortants to mammals.

**Results:**

Our results demonstrated that most of the 15 isolates were reassortants and generated four novel genotypes (B62-B65), which incorporated the gene segments from Eurasian H9N2 lineage, North American H9N2 branch, and H5N1 viruses. It was noteworthy that the newly identified genotype B65 has been prevalent in China since 2007, and more importantly, different H9N2 influenza viruses displayed a diverse pathogenicity to mice. The isolates of the 2008-2010 epidemic (genotypes B55 and B65) were lowly infectious, while two representative viruses of genotypes B0 and G2 isolated from the late 1990s were highly pathogenic to mice. In addition, Ck/SD/LY-1/08 (genotype 63, containing H5N1-like NP and PA genes) was able to replicate well in mouse lungs with high virus titers but caused mild clinical signs.

**Conclusion:**

Several lines of evidence indicated that the H9N2 influenza viruses constantly change their genetics and pathogenicity. Thus, the genetic evolution of H9N2 viruses and their pathogenicity to mammals should be closely monitored to prevent the emergence of novel pandemic viruses.

## Background

H9N2 influenza viruses are panzootic in birds worldwide. Statistical analysis of the host range and location of H9N2 subtype influenza A viruses in the NCBI database according to HA gene revealed that approximately 60% of all the H9N2 influenza viruses were isolated from chickens, with the remainder from wild birds (16.8%), ducks (8.9%), turkeys (6.7%), and other domestic avian populations (3.7%) (data not shown). In addition, the overwhelming majority (94.2%) of H9N2 influenza viruses were isolated in Asia, with > 65% coming from mainland and Hong Kong of China (data not shown). Since in China the H9N2 virus was first time isolated in 1994 while approximately 74 different genotypes have been observed till now and new lineages and genotypes continuously identified throughout China [1]. Two main distinct lineages of H9N2 influenza viruses represented by A/Chicken/Beijing/1/94 (Ck/Bei-like) and A/Quail/Hong Kong/G1/97 (G1-like) have become endemic in China since the mid-1990s [2-5]. The G1-like viruses were mainly detected in quail of southern China [3,4]. While, the Ck/Bei-like viruses were first prevalent among chickens, ducks, and other minor poultry species in both southern and northern China [4,5] and then were gradually replaced by the F98-like (represented by A/Chicken/Shanghai/F/98) viruses since 2000 onward [5,6]. These viruses evolved from the Ck/Bei-like lineage but replicated and transmitted more effectively than the antecedent viruses in chickens under experimental conditions [5]. Recent studies revealed that most of the viruses originated from the Ck/Bei-like viruses and formed multiple genotypes through complicated reassortments with G1-like, G9-like (represented by A/Chicken/Hong Kong/G9/97), Y439-like (represented by A/duck/Hong Kong/Y439/97), and F98-like viruses [2].

In order to systematically analyze the evolution of H9N2 influenza viruses, the genealogy of H9N2 viruses was further studied. In early reports, the methods of genealogy classification were mainly determined on the basis of genetic relationship of all the eight gene segments with representative strains, such as Ck/BJ/1/94, Ck/SH/F/98, Qa/HK/G1/97 [2,7]. Later, the H9N2 influenza viruses isolated in southern China were classified into A (viruses with a G1-like HA) and B (viruses with a Ck/Bei-like HA) genotype series with different sources and gene constellations [3,4]. On these basis, the H9N2 viruses were further divided into seven different series (A ~ G) according to their HA lineages. And in each series, different genotypes were designated sequentially by additional 0, 1, 2 and so on, according to their systematic nomenclature [1,3,4]. For example, in genotype B series, non-reassortment Ck/Bei-like viruses were designated as B0, while reassortment Ck/Bei-like viruses were designated sequentially as B1, B2, and so on, according to when the novel genotype was first identified [1]. The nomenclature system systematically unified the lineages and genotypes of H9N2 influenza viruses, which revealed the phylogenetic diversity and genotypic complexity of H9N2 influenza viruses worldwide [1].

The high evolution rates with complicated reassortments prompted that H9N2 virus may threat to human health by genetic contribution to the generation of novel pandemic influenza virus. A recent study highlighted the importance and risk of H9 reassortants between avian H9N2 virus and the pandemic H1N1/2009 influenza virus exhibited higher pathogenicity to mice than both the parental viruses [8]. In fact, H9N2 viruses have sporadically crossed the barrier from birds to mammals and several recently emerging F98-like viruses were suspected to have high pathogenicity to mice [9]. Noticeably, H9N2 virus has caused several human infections with mild respiratory disease in Hong Kong and mainland of China since 1997 [10]. Moreover, some H9N2 viruses isolated from live bird markets in Hong Kong, with Leu (L) residue at amino acid position 226 (numbered by H3) in hemagglutinin (HA) receptor binding site (RBS), displayed human virus-like receptor specificity [11]. Further studies revealed that the Leu226 (numbered by H3) in HA gene was found to be important for the transmission of the H9N2 viruses in ferrets, and mixing the surface glycoproteins of an H9N2 virus with the internal genes of an human H3N2 virus resulted in enhanced replication and efficient direct transmission in ferrets [12]. These results raised great concerns about viral evolution and suggested that the H9N2 avian virus could be of pandemic importance.

## Results

### Phylogenetic analysis

Eight gene segments of the 15 isolates were phylogenetically analyzed together against the full length sequences of 379 representative viruses in China. The phylogenetic tree of HA gene segments demonstrated that the Ck/Bei-like was the predominate lineage and could be separated into four sub-lineages in China (Additional file 1, Figure.S1A). The G9-like sub-lineage and the F98-like sub-lineage were prevailing around the year 2000. Whereas the JS98-like viruses (represented by A/ckichen/Jiangsu/1/98) circulated from 2000 to 2005, and the Y280-like sub-lineage (represented by A/duck/Hong Kong/Y280/97), which composes the majority of H9N2 influenza viruses, has circulated in the recent 5 years (Additional file 1, Figure.S1A). In the present study, the 13 viruses isolated from 2007 to 2010 fell into the Y280-like sub-lineage, and Ck/SD/WF/98 belonged to the G9-like cluster (Table 1 and Additional file 1, Figure.S1A). Interestingly, the Ck/HLJ/u/98 isolate displayed high identity (99.4%) with Ck/HLJ/35/00 [Genbank: DQ064366], which belonged to the Ty/WI/1/66-like North American lineage (Additional file 1, Figure.S1A). A similar phylogenetic structure was seen in NA gene, but in contrast to HA, the NA gene of the Ck/Bei-like lineage could be divided into three major subgroups: F98-, G9-, and Y280-like (Additional file 1, Figure.S1B). Most of the isolates were in the Y280-like sub-lineage, while Ck/SD/lx929/07, Ck/SD/lx1023/07 and Ck/HLJ/u/98 grouped with G9-like viruses (Table 1 and Additional file 1, Figure.S1B), which did not contain a stalk deletion (Additional file 2, Table S1). It was also noteworthy that a human H9N2 isolate A/Guangzhou/333/99 [Genbank: AY043024] shared high identity (98.3%) and formed a sister cluster with Ck/HLJ/u/98 (Additional file 1, Figure.S1B).

**Table 1:** Gene constellations of different genotypes of the H9N2 AIVs


**Virus**	**Genotype**	Genetic source*							
		
		**PB2**	**PB1**	**PA**	**HA**	**NP**	**NA**	**M**	**NS**

Ck/SD/WF/98	B0	BJ94	BJ94	BJ94	G9	BJ94	Y280	BJ94	BJ94
Ck/HLJ/u/98	G2	G1	G1	G1	Nor A	Nor A	G9	BJ94	SH96
Ck/SD/lx929/07	B62	F98	BJ94	Y439	Y280	F98	G9	BJ94	SH96
Ck/SD/lx1023/07	B62	F98	BJ94	Y439	Y280	F98	G9	BJ94	SH96
Ck/SD/LY-1/08	B63	F98	BJ94	H5N1	Y280	H5N1	Y280	BJ94	SH96
Ck/SD/BD/08	B55	F98	F98	F98	Y280	F98	Y280	G1	F98
Ck/SD/KD/09	B55	F98	F98	F98	Y280	F98	Y280	G1	F98
Ck/SD/02/08	B64	F98	BJ94	F98	Y280	F98	Y280	G1	F98
Ck/SD/01/09	B65	H	F98	F98	Y280	F98	Y280	G1	F98
Ck/SD/02/09	B65	H	F98	F98	Y280	F98	Y280	G1	F98
Ck/SD/H/09	B65	H	F98	F98	Y280	F98	Y280	G1	F98
Ck/SD/BD/10	B65	H	F98	F98	Y280	F98	Y280	G1	F98
Ck/SD/01/10	B65	H	F98	F98	Y280	F98	Y280	G1	F98
Ck/SD/02/10	B65	H	F98	F98	Y280	F98	Y280	G1	F98
Ck/SD/03/10	B65	H	F98	F98	Y280	F98	Y280	G1	F98

* PB, polymerase basic protein; PA, polymerase acidic protein; HA, hemagglutinin; NP, nucleocapsid protein; NA, neuraminidase; M, matrix; NS, nonstructural; BJ94, Ck/Bei-like (represented by A/Chicken/Beijing/1/94); Y280, Y280-like (represented by A/duck/Hong Kong/Y280/97); F98, F98-like (represented by A/Chicken/Shanghai/F/98); G1, G1-like (represented by A/Quail/Hong Kong/G1/97); G9, G9-like (represented by A/Chicken/Hong Kong/G9/97); Y439, Y439-like (represented by A/duck/Hong Kong/Y439/97); SH96, SH96-like (represented by A/Quail/Shanghai/8/96); H, unknown source and named as H-like (represented by A/chicken/Shandong/H/09); H5N1, H5N1-like.

The M genes of Ck/Bei-like and G1-like lineages were predominant in H9N2 viruses (Additional file 1, Figure.S1C). And the G1-like M gene was more prevalent than that of other gene segments and was clearly separated into three sub-lineages among different periods of time (Additional file 1, Figure.S1C). Ten of the eleven 2008-2010 isolates fell into the same G1-like lineage, while the other five isolates grouped into the Ck/Bei-like branch (Table 1 and Additional file 1, Figure.S1C). The phylogenetic tree of NS gene displayed that all the 15 isolates grouped into the Ck/Bei-like lineage, which predominated in China and generated three sub-lineages (Additional file 1, Figure.S1D). Most of the 2008-2010 isolates belonged to the F98-like group, while except Ck/SD/WF/98 was in the Ck/Bei-like cluster, the other four viruses clustered into the SH96-like group, which represented by A/Quail/Shanghai/8/96 (Table 1 and Additional file 1, Figure.S1D).

Concerning the PB2 gene, Chinese H9N2 viruses mainly fell into three different lineages: Ck/Bei-, F98-, and G1-like (Additional file 1, Figure.S1E). Interestingly, seven isolates together with other six 2007-2010 viruses from Genbank, formed a novel independent lineage that originated from an unknown source and were genetically adjacent to the G1-like cluster (named as H-like lineage, represented by A/chicken/Shandong/H/09) (Table 1 and Additional file 1, Figure.S1E). A/swine/Yangzhou/1/2008(H9N2) [Genbank: HM998919] also located into this new cluster (Additional file 1, Figure.S1E). Analysis of the PB1 gene revealed that China H9N2 viruses could also be separated into three major lineages. The viruses isolated since 2008 in this study mainly belonged to the F98-like lineage, while other early isolates clustered into the Ck/Bei-like lineage, and the Ck/HLJ/u/98 belonged to the G1-like lineage (Table 1 and Additional file 1, Figure.S1F).

The topologies of the PA and NP gene segments were similar with the PB1 gene, and the majority of the 15 H9N2 isolates located in the F98-like lineage. For the PA gene, most of the isolates from 2008 to 2010 clustered in the F98-like group, while two early isolates (Ck/SD/WF/98 and Ck/HLJ/u/98) fell into the Ck/Bei-like and G1-like lineages, respectively. Two isolates, Ck/SD/lx929/07 and Ck/SD/lx1023/07, belonged to the Y439-like lineage, which was an established H9N2 lineage originating from H5N1-like virus (Additional file 1, Figure.S1G). Importantly, Ck/SD/LY-1/08 contained H5N1-origined PA and NP genes. The NP genes of the H9N2 isolates mainly grouped with the F98-like viruses. However, the NP genes of Ck/HLJ/u/98 and Ck/SD/WF/98 belonged to the Ty/WI/1/66-like North America lineage and the Ck/Bei-like lineage, respectively (Table 1 and Additional file 1, Figure.S1H).

### Genotype analysis

**Figure 1:**
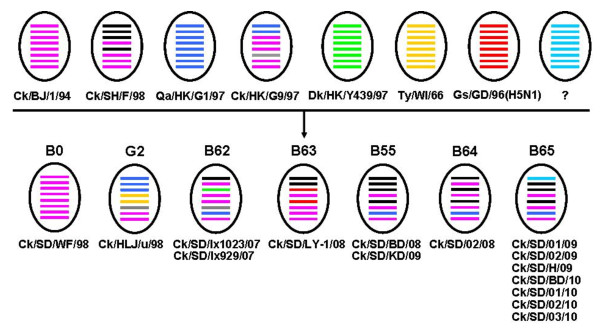
**Genotypes of H9N2 influenza viruses isolated from chickens in northern China**. The seven different genotypes are designated B0, G2, B62, B63, B55, B64 and B65. The eight horizontal bars in ovals represent eight gene segments of AIV (from top to bottom): PB2, PB1, PA, HA, NP, NA, M, and NS. Each color represents a virus lineage. "?", displays an unknown source lineage.

In the present study, the 15 H9N2 isolates were further classified into seven genotypes according to the systematic and effective methods of genotype classification defined in previous studies [1]. Six genotypes were Ck/Bei-like (genotype B0, B55, B62, B63, B64 and B65), and one genotype was Ty/WI/1/66-like (genotype G2) (Figure 1, Table 1). Ck/SD/WF/98 was a typical Ck/Bei-like virus with non-reassortment and therefore classified into the genotype B0 group. Two isolates (Ck/SD/BD/08 and Ck/SD/KD/09) originated from the F98-like lineage and the M genes came from the G1-like lineage, belonged to genotype B55 which contains viruses frequently isolated in recent years [9,13].

Four novel H9N2 genotypes identified in this study were designated as B62, B63, B64 and B65. Genotype B62 viruses (Ck/SD/lx929/07 and Ck/SD/lx1023/07) were quintuple reassortments with Ck/Bei-, F98-, G9-, Y439-like and SH96-like viruses, which originated from Ck/Bei-like and had F98-like PB2 gene, G9-like NA gene, SH96-like NS gene, and PA gene from the Y439-like lineage; The Ck/SD/LY-1/08 isolate was classified into genotype B63, which came from Ck/Bei-like virus and contained H5N1-like PA and NP gene segments; The Ck/SD/02/08 isolate had the same genetic source with genotype B55 except the PB1 gene came from Ck/Bei-like lineage, was classified into genotype B64. Seven gene segments of genotype B65 viruses (Ck/SD/01/09, Ck/SD/02/09, Ck/SD/H/09, Ck/SD/BD/10, Ck/SD/01/10, Ck/SD/02/10, Ck/SD/03/10) were identical to genotype B55, but their PB2 gene was derived from an unknown origin.

### Molecular analysis

The deduced HA, NA, and PB2 amino acid sequences of the 15 viruses were respectively compared with those of representative viruses. Interestingly, the HA gene of Ck/SD/lx929/07 had a 21-nucleotide deletion from 1267 to 1287 encoding 7 amino acids, while another virus (Ck/SD/lx1023/07) isolated from the same poultry farm at the same time did not contain a deletion in its HA protein. This deletion located in the HA2 domain and did not affect any potential glycosylation site of HA (Additional file 2, Table S1). There were seven conserved potential glycosylation sites in the HA proteins of most H9N2 viruses (amino acid positions 29-31, 141-143, 218-220, 298-300, 305-307, 492-494, and 551-553, H9 numbering). The addition and deletion of the potential glycosylation sites in the present isolates were shown in Additional file 2, Table S1, but whether the change in the glycosylation sites might affect the viral characteristics needed to be explored further. Moreover, most of the H9N2 viruses tested in this study had the same P-A-R-S-S-R ↓ G amino acid motif at the cleave site (arrow) (Additional file 2, Table S1), which was characteristic of H9N2 virus from land-based poultry [7]. However, Ck/HLJ/u/98 had a P-A-V-S-S-R ↓ G motif, i.e., contained a Val instead of an Arg at position 335 (H9 numbering) (Additional file 2, Table S1).

The RBS in HA protein associates with the receptor binding specificity, and determines the infection spectrum of influenza virus. Ten of the fifteen characterized viruses had the human-like motif Leu226 (H3 numbering), and four isolates contained the avian-like motif Gln226 in the RBS (Additional file 2, Table S1). Interestingly, almost all of the Ck/BJ/1/94-like viruses had a RBS-related Ala-to-Val/Thr mutation at position 190 (H3 numbering) (Additional file 2, Table S1).

It was also well know that the amino acids at position 627 and 701 of the PB2 gene (H3 numbering) were recognized as the critical mammalian host determinant. All the present H9N2 isolates contained the Glu (E) and Asp (D) in the two sites, respectively (Additional file 2, Table S1), which represented the weak virulence in mammalian host. Furthermore, the length of NA stalk may affect the pathogenicity of influenza virus, and most of the H9N2 isolates had the same "marking" deletion of three amino acids (positions 63-65) at the NA stalk region, except for three isolates (Ck/SD/WF/98, Ck/SD/lx929/07, and Ck/SD/lx1023/07). The analysis showed that the diverse molecular characters existed in H9N2 viruses, such as RBS, amino acid motif at the cleave site, and deletion in HA or NA protein, but the significance of the diversity should be further studied.

### Pathogenicity to mice

**Figure 2:**
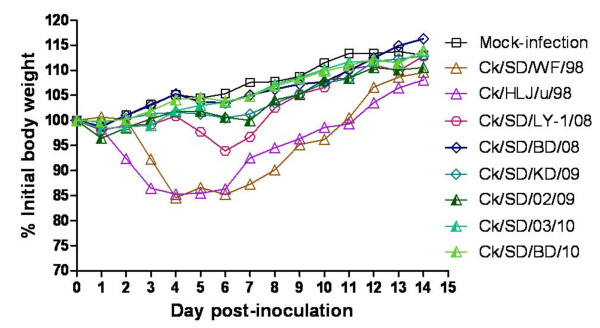
**Mean changes in body weight of mice infected with H9N2 viruses**. BALB/c mice were inoculated i.n. with each of eight H9N2 viruses at a dose of 10^7 ^EID_50_. The body weights were monitored daily for a 14-day observation period and expressed as a percentage of the initial value. The data represents the mean of four mice in each group.

**Figure 3:**
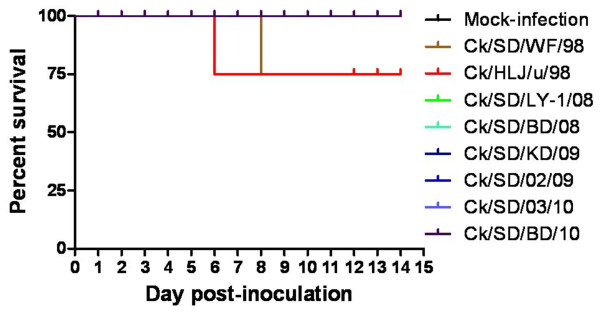
**Survival percentage of mice infected with H9N2 viruses**. Groups of mice (n = 4) were inoculated i.n. with each of eight H9N2 viruses at a dose of 10^7 ^EID_50_. The percent survival was observed daily for 14 days.

To investigate the adaptation and potential pathogenicity of different H9N2 genotypes to mammals, we selected eight representative isolates from different genotypes with good reproductive capacity in SPF eggs (Table 2) and then these viruses were inoculated intranasally (i.n.) into the exposure groups of 6-week-old female BALB/c mice. The onset and the duration of clinical symptoms induced by representative H9N2 isolates at the dose of 10^7 ^EID_50 _were as follows: five isolates from 2008 to 2010 (genotype B55 and B65) caused no gross lesions in lungs of the inoculated mice at 3 days post-infection (d.p.i.), as the mice remained healthy and gained body weight during the infection (Figure 2, Table 3). In contrast, the mice inoculated with Ck/SD/WF/98 (genotype B0) or Ck/HLJ/u/98 (genotype G2) started showing inactivity, ruffled fur, and obvious body weight loss at 2 and 3 d.p.i. (Figure 2 and Table 3). From 4 to 6 d.p.i., the mice of the two infectious groups exhibited severe inappetence, emaciation, and the most significant weight loss (14.77 and 14.72% for the Ck/SD/WF/98- and Ck/HLJ/u/98-inoculated groups, respectively) (Figure 2 and Table 3). The inappetence and inactivity were correlated with a gradual loss of body weight, and death was observed after inoculation of mice with Ck/SD/WF/98 or Ck/HLJ/u/98 viruses (Figure 3). Another strain, Ck/SD/LY-1/08 (genotype 63) displayed milder clinical signs and less weight loss (the maximum weight loss was 6.09%) compared to the Ck/SD/WF/98 and Ck/HLJ/u/98 strains (Figure 2 and Table 3). The survived mice clinically recovered from infection at 7 d.p.i., and their body weight reached the same level as the mock-infected control groups at 14 d.p.i. (Figure 2).

**Table 2:** Reproductive capacity of the representative H9N2 AIVs in embryonated eggs


**Virus**	**HA**	**EID_50_(log_10_/0.1 ml)**	**ELD_50_(log_10_/0.1 ml)**	**Genotype**

Ck/SD/BD/08	2^7^	7.75	5.0	B55
Ck/SD/KD/09	2^8^	7.75	5.25	B55
Ck/SD/BD/10	2^11-12^	8.75	< 7.5	B65
Ck/SD/03/10	2^9^	8.75	8.5	B65
Ck/SD/02/09	2^9~10^	8.25	6.75	B65
Ck/SD/LY-1/08	2^11~12^	8.5	8.0	B63
Ck/HLJ/u/98	2^9~10^	8.0	< 6.5	G2
Ck/SD/WF/98	2^9~10^	7.75	7.5	B0

**Table 3:** Differences in pathogenicity among the H9N2 AIVs to BALB/c mice


**Virus**	**Virus replication in mouse lung at 3 d.p.i. (log_10_EID_50_/0.1ml)**	**Clinical syndromes**		**Seroconversion** **(HI mean titer)**
			
		**Body weight (%)^c^**	**Fur**	

Ck/SD/BD/08	1/3 (<^a^)	gain (16.27)	unruffled	< 10
Ck/SD/KD/09	1/3 (<^a^)	gain (12.86)	unruffled	20-160
Ck/SD/BD/10	1/3 (<^a^)	gain (13.97)	unruffled	40-320
Ck/SD/03/10	-^b^	gain (13.82)	unruffled	10-20
Ck/SD/02/09	2/3 (1.5, 2.75)*	gain (10.55)	unruffled	160-320
Ck/HLJ/u/98	3/3 (2.5, 3.75, 2.5)**	lose (14.72)	ruffled	< 10
Ck/SD/LY-1/08	3/3 (5.0, 5.5, 5.25)**	lose (6.09)	Little ruffled	20-40
Ck/SD/WF/98	3/3 (1.5, 2.75, 2.0)**	lose (14.77)	ruffled	40-80
Mock-infected control	-	gain (13.04)	unruffled	-

^a ^<, Virus titer < 1 log_10_EID_50_.

^b ^-, No virus or haemagglutination inhibition (HI) titer was detected.

^c ^The percentage of the mean maximum weight loss or gain among the inoculated mice over the course of infection.

* The data were compared with the Ck/SD/BD/08 group by 2-way ANOVA, respectively, *P < 0.01, **P < 0.001.

The replication ability of the six H9N2 isolates in mice was evaluated by the virus titer in the lungs of each infected mice group at 3 d.p.i. (Table 3). Four strains replicated well in mice lungs without prior adaptation, among which Ck/SD/WF/98 (genotype B0), Ck/HLJ/u/98 (genotype G2), and Ck/SD/02/09 (genotype 65) were able to replicate well in mice with different virus titers at 3 d.p.i. (Table 3). And mice inoculated with Ck/SD/LY-1/08 had a much higher virus titer (5.0, 5.5 and 5.25 log_10_EID_50_/0.1 ml) than those inoculated with other strains (1.5 to 2.75 log_10 _EID_50_/0.1 ml) at 3 d.p.i. (Table 3).

**Figure 4:**
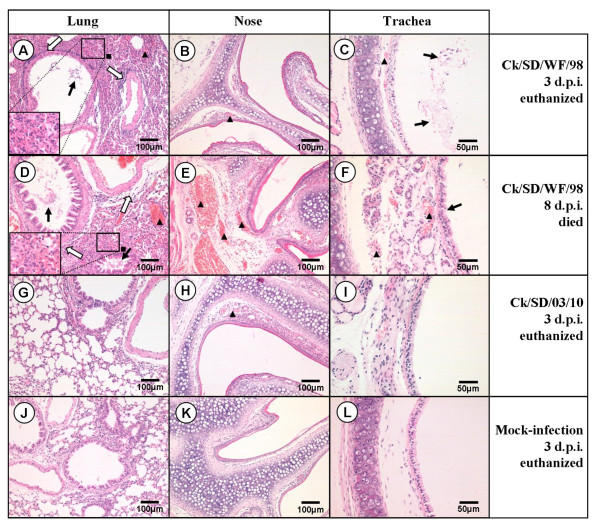
**Representative histopathological changes in Hematoxylin and Eosin (H&E)-stained respiratory system tissues (nose, trachea, and lung)**. Ck/SD/WF/98 virus-infected mice displayed severe bronchopneumonia and interstitial pneumonia in lung tissues (A and D), which showed interstitial edema and thickening of the alveolar walls, alveolar lumen flooded with dropout of alveolar cells, erythrocytes, and inflammatory cells (black square), bronchial epithelial cell desquamation (thick solid arrow) and extensive lymphocyte, neutrophil, and plasma cell infiltrates around the bronchiolitis and blood vessels (thick white arrow); congestion in the blood vessels (black triangle); Light (B, H) and intense (E) congestion in the blood vessels of the nasal submucosa caused by the Ck/SD/WF/98 and Ck/SD/03/10 viruses, respectively (black triangle); Congestion in the blood vessels of the tracheal submucosa (black triangle) and dropout of the mucous epithelium in the trachea (thick solid arrow) caused by the Ck/SD/WF/98 virus (C and F). There were no obvious histopathological changes in the respiratory system tissues of the Ck/SD/03/10-infected group and no significant difference compared to the PBS mock-infection group.

The damage to respiratory tissues of the infected mice was consistent with the clinical symptoms and lung virus titers. Ck/SD/WF/98 (genotype B0)- and Ck/HLJ/u/98 (genotype G2)-infected mice displayed severe bronchopneumonia and interstitial pneumonia (Figure 4). Ck/SD/LY-1/08 (genotype 63)-infected mice also suffered heavy bronchopneumonia and a higher virus titer in their lungs, although it caused less severe clinical syndromes, with 6.09% of the maximum weight loss and little ruffled fur (Table 3 and Figure 2). Additionally, there were no obviously tissue lesions in the respiratory system among the other five isolates in genotype 55 or 65 (i.e., Ck/SD/02/09, Ck/SD/BD/10, Ck/SD/03/10, Ck/SD/KD/09, and Ck/SD/BD/08). The pathogenicity test of the representative H9N2 viruses was repeated twice in mice and the similar results were obtained, which indicated that the different H9N2 viruses in our study possessed diverse replication ability and virulence in mice.

## Discussion

H9N2 viruses have undergone extensive reassortments to generate multiple reassortants and genotypes [1-7,9,13,14]. In the present study, extensive sequence data was used to characterize the evolutionary pattern by genetic and pathogenic diversity of the H9N2 influenza viruses in China. Phylogenetic analysis indicated that both Ck/Bei-like and F98-like viruses co-circulated in China from 1998 to 2010 (Additional file 1, Figure.S1). The internal gene segments of most viruses isolated after 2008 were derived from the F98-like group, indicating that F98-like has been the dominant lineage in recent years. Phylogenetic analysis also indicated multiple sub-lineages in each gene segments were well-separated and evolved independently, which indicated that each gene segment of the H9N2 viral genome displayed a high evolutionary rate.

Multiple reassortants and an increasing number of H9N2 influenza virus genotypes have been continuously identified throughout China in recent years, although most of the viruses were transients and could not be established in avian hosts [1,4,5]. In the present study, all of the representative strains isolated from diseased chicken in northern China were reassortants exception Ck/SD/WF/98 (which was a pure Ck/Bei-like strain) (Table 1 and Additional file 1, Figure.S1). One novel stable and predominant lineage (genotype B65) was identified in our study. This triple-reassortant lineage possessed a G1-like M gene segment, a PB2 gene with an unknown origin, and other gene segments from F98-like viruses (Table 1 and Additional file 1, Figure.S1). Together with our 7 isolates, 13 H9N2 viruses have so far been detected in this genotype which were mainly isolated from chickens in northern and southeastern China from 2007 to 2010 (Additional file 1, Figure.S1E). Interestingly, A/swine/Yangzhou/1/2008 (isolated from swine) also belonged to this new genotype (Additional file 1, Figure.S1E), indicating that genotype B65 virus has been established in chickens in different regions of China and has spread to mammals. Other new identified genotypes were composed of one or two strains (Table 1, Figure 1). However, further sequence data collection is needed to confirm if they are transient reassortants or have been established in avian.

H9N2 influenza viruses can also generate novel reassortment or genotype viruses that carry gene segments with wide evolutionary distances. It has been reported that H9N2 strain A/swine/Korea/S190/2004 was a reassortant between a Eurasian lineage and a North American lineage viruses [1]. In China, Ck/HLJ/35/00 has only been reported as H9N2 virus that possesses HA [Genbank: DQ064366] and NP [Genbank: DQ064447] genes from an early North American lineage (represented by Ty/WI/1/66) [1,2]. Interestingly however, the Ck/HLJ/u/98 isolate analyzed in our study possessed each of eight gene segments with the highest homology to those of Ck/HLJ/35/00 (Additional file 1, Figure.S1). These findings suggested that the two strains might have the same origin, and this genotype was not transient rather circulated in China for several years.

The novel H9N2 reassortants or genotypes with wide evolutionary distances not only generated by hybridization among different lineages of H9N2 viruses, but also by reassortment between H9N2 and H5N1 viruses. The first reported evidence of hybridization between H9N2 and H5N1 was the case of human infected by highly pathogenic avian influenza (HPAI) H5N1 virus in 1997, and the Qa/HK/G1/97(H9N2)-like viruses were hypothesized to have been involved in the generation of the H5N1 virus [15]. Previously isolated H9N2 influenza viruses that reassorted with H5N1 viruses could also be found elsewhere: those possessing the H5N1-like PB1 gene segments were from southeastern China [14], and H9N2 viruses with NS genes originating from the HPAI H5N1 lineage were isolated from Pakistan [16]. However, the pathogenicity of these reassortants was not clear [14,16]. Noticeably, in our study, both the PA and NP genes of Ck/SD/LY-1/08 (genotype 63) shared high homology (98%-99%) with the H5N1 viruses circulating around the year 2005, such as A/chicken/China/1/02 (H5N1) [GenBank: DQ023146], A/swine/Shandong/2/03 (H5N1) [GenBank: AY646426, AY700213], and A/environment/Qinghai/1/2008 (H5N1) [GenBank: FJ455823, FJ455825]. Interestingly, one H5N1 reassortment virus, A/plateau pika/Qinghai/04/2007 (H5N1), was closely related to Ck/SD/LY-1/08 in its PB2 (98.4% homology) [GenBank: FJ390058], PA (98.5% homology) [GenBank: FJ390060], and NP (99.6% homology) [GenBank: FJ390062] gene segments (Additional file 1, Figure.S1E, G and H). It also contained the Ck/Bei-like M and NS genes, as well as a Y439-like PB1 gene. It was demonstrated that multiple reassortments have occurred between H9N2 and H5N1 subtypes since the early outbreaks, and they continued exchanging internal gene segments and generating novel viruses.

H9N2 viruses of different genotypes and reassortment patterns could have huge differences in their pathogenicity and transmission in mammals (BALB/c mice and swine) under experimental conditions [2,7,9]. In the present study, the majority of the recently circulated genotype B65 and B55 viruses, which include the PB2 gene segment from an unknown origin and/or the M gene segment from the G1-like lineage, displayed low infective ability in mice (Table 3 and Figure 2). In addition, Ck/SD/LY-1/08 (genotype B63) was able to replicate well in mice lungs with high virus titer but caused mild clinical symptoms (Table 3 and Figure 2). Natural selection suggests that a less virulent strain is more likely to co-exist with the host population because mobile, living hosts will transmit the strains most effectively [17]. Thus, whether the changes in the host adaptation and replication ability of Ck/SD/LY-1/08 are associated with the H5N1 donor of the PA and NP gene segments must be explored further. Importantly, two early strains in our study (Ck/HLJ/u/98 in genotype G2 and Ck/SD/WF/98 in genotype B0) caused the death of infected mice without prior adaptation (Figure 3), though a previous study of several H9N2 viruses belonging to the same genotypes reported non-lethality in mice [2]. The similar discordant infectivity in mice with same genotype viruses was also observed in this study, Ck/SD/BD/10, Ck/SD/03/10 and Ck/SD/02/09 were all in the genotype B65, and the former two viruses nearly couldn't replicate in mice lungs, but the third strain were able to replicate well in mice lungs without prior adaptation. It was reported that the mutations of E627K and D701N in PB2 gene were the key factors for a virus to acquire the ability to adapt to increase virulence in a new mammalian host [18]. Molecular analysis demonstrated that all of the viruses in our study possessed the conservative residues Glu627 and Asp701 in PB2 gene. Therefore, it is probable that the virulence of H9N2 influenza viruses is not fully relevant to whole-gene homology, and some amino-acid mutations or deletions may dramatically alter the virulence of influenza viruses.

In light of the persistent evolution of H9N2 viruses with high evolution rate, it is highly necessary to monitor the evolution and evaluate the virulence of novel avian H9N2 viruses to mammal animals, which may discover and prohibit the potential threat to human by novel viruses, and provide the key clues for preventing new pandemic influenza.

## Conclusions

Here, we described the genetic and pathogenic characteristics of the representative H9N2 viruses isolated from chicken farms in northern China over a 12-year span. Phylogenetic analyses revealed that H9N2 influenza viruses evolved quickly in China, and new genotypes were frequently generated in chicken flocks. The present study identified two stable H9N2 lineages (genotype B55 and B65) that have been circulating in China in recent years. In addition, different H9N2 viruses possessed diverse replication capacity and pathogenicity in mice.

In summary, our study provides evidences to further understand the genetic evolution and inter-species transmission of H9N2 influenza A viruses. Their different pathogenicitiy behavior and host adaptations to mice may likely be associated with both the gene reassortments and key amino acid mutations. Thus, surveillance followed by large-scale sequencing efforts and animal experiments are critically important to detect the virulent variants and provide insight into the key features for emergence of pandemic viruses.

## Materials and methods

### Virus isolation and identification

Surveillance was conducted in northern China from 1998 to 2010. Tracheal, cloacal and fecal swabs, and lung tissues were collected from sick or dead chickens from chicken farms. The tissue homogenates of clinical specimens were centrifuged for 10 minutes at 8, 000 × g, and after removing bacterial contaminants by micropore membrane filters (0.22 μm) the supernatant was incubated in the allantoic cavities of 10-day-old specific pathogen free (SPF) chicken eggs (Beijing Merial Vital Laboratory Animal Technology Company) at 37°C for 72 h for viral isolation, finally the allantoic fluids was harvested and stored at -80°C. The purification of all the strains was done by the end-point dilution assay [19], and subtyping was performed as previously described [20]. Fifteen avian H9N2 viruses were chosen for detailed analysis (Additional file 3, Table S2).

### Viral gene sequencing

Viral RNA was extracted from infected allantoic fluids using Trizol reagent (Invitrogen). Reverse transcription (RT) was performed by using the Uni12 primer (AGCAAAAGCAGG), and specific primers were designed amplifying the eight full-length gene segments of the virus. The PCR products were purified and sequenced from the Beijing Genomics Institute using synthetic oligonucleotides.

### Genetic and phylogenetic analyses

To determine the molecular evolutionary characteristics of H9N2 viruses isolated in China, eight gene segments from each of 15 isolates were phylogenetically analyzed together with all the full length sequences of the representative viruses of China available in GenBank. The nucleotide and amino acid sequences of each gene segment were analyzed by DNAMAN (Version 5.2.2, Lynnon Biosoft, USA) and DNASTAR (DNASTAR, Inc., Madison, MI, USA) softwares. The nucleotide sequence-based phylogenetic trees were generated with MEGA 4.1 http://www.megasoftware.net via Neighbor-Joining algorithm and the reliability of the trees was assessed by bootstrap analysis with 1, 000 replications.

### Genotype analysis

Genotype analysis was performed systematically for each of the eight gene segments based on the distribution of lineages in phylogenetic trees, and the genes sharing over 95% homology in the same lineage were considered as one genotypic group [1]. H9N2 viruses according to their HA lineages were divided into seven series, designated as A~G. Viruses with G1-like, Ck/Bei-like, Ck/Korea/38349-p96323/96-like, Y439-like, Dk/HK/289/78-like, Qa/HK/AF157/92-like, Ty/WI/1/66-like HA genes were classified into genotype A, B, C, D, E, F, and G series, respectively [1]. In each of the series, different genotypes were designated sequentially by additional 0, 1, 2 and so on, according to different gene constellations with the systematic nomenclature [1,3,4].

### Pathogenicity test in mice

H9N2 influenza viruses from each genotype were selected to assess their potential pathogenicity to mammal. BALB/c mice (6-week-old, female) were purchased from Vital River Laboratories, Beijing. Mice in each group (n = 7) were lightly anesthetized with Zoletil (tiletamine-zolazepam; Virbac; 25 μg/g) and inoculated i.n. with 10^7 ^EID_50 _of H9N2 virus in a volume of 50 μl. Mock-infected control mice were inoculated i.n. with 50 μl phosphate-buffered saline (PBS). Three mice were humanely euthanized at 3 d.p.i., and their lungs were collected, ground and homogenized in cold PBS under sterile conditions. Then the solid debris was pelleted by centrifugation at 5, 000 × g for 10 min, and the homogenates were used for virus titrations in 10-day-old embryonated chicken eggs. Virus titers were reported in units of log_10_EID_50 _per 0.1 ml. Nose, trachea, and part of left lung lobes were fixed in 10% buffered formalin, embedded in paraffin, and the tissues were stained with hematoxylin-eosin for histopathological evaluation. The rest of the mice were monitored daily for general behavior and clinical signs, including food intake, body weight, inactivity, and mortality, for 14 days.

### Ethics Statement

The use of all laboratory animals and animal subjects in this study was approved by the Beijing Association for Science and Technology, with approval ID SYXK (Beijing) 2007-0023, and followed by the Beijing Laboratory Animal Welfare and Ethical Guidelines of the Beijing Administration Committee of Laboratory Animals.

### Nucleotide sequence accession numbers

The nucleotide sequences of 15 H9N2 influenza viruses isolated in this study have been submitted to the GenBank database and have assigned the accession numbers (JF795035-JF795148).

## Competing interests

The authors declare that they have no competing interests.

## Authors' contributions

This study was elaborated and launched by YB, LS and WL, who leading the research groups on virology and genetics. YB and LL accomplished the virus sequencing, phylogenetic, molecular analyses and the animal experiments. YB, ZQ, YY, JL, YZ, HG and JhL did much effort on virus collection and isolation. YB, LL and BZ participated in the work of manuscript preparation and revision. All authors have read and approved the final manuscript.

## Supplementary Material

Additional file 1 **Figure.S1**. Unrooted neighbor-joining phylogenetic trees for the HA (34-1573 nt) (A), NA (20-1387 nt) (B), M (26-1007 nt) (C), NS (27-849 nt) (D), PB2 (28-2289 nt) (E), PB1 (25-2242 nt) (F), PA (25-2175 nt) (G), and NP (46-1526 nt) (H) genes of H9N2 influenza A viruses. Trees were generated by the Maximum Composite Likelihood model of Neighbor-Joining algorithm with MEGA 4.1 http://www.megasoftware.net. The reliability of the trees was assessed by bootstrap analysis with 1, 000 replicates and only bootstrap values ≥ 90% were shown. Different lineages were indicated by different colors. The viruses obtained in the present study were marked with red circles, and the representative strains in each lineage were marked with yellow squares. Click here for file

Additional file 2 **Table S1**. Comparison of amino acid sequences of HA, NA, and PB2 genes of representative H9N2 viruses from northern China. Click here for file

Additional file 3 **Table S2**. The clinical information of representative avian H9N2 influenza viruses isolated from northern China. Click here for file

## References

[B1] Dong G Luo J Zhang H Wang C Duan M Deliberto TJ Nolte DL Ji G He H Phylogenetic Diversity and Genotypical Complexity of H9N2 Influenza A Viruses Revealed by Genomic Sequence Analysis PLoS One 2011 6 e17212 10.1371/journal.pone.0017212 21386964 PMC3046171

[B2] Li C Yu K Tian G Yu D Liu L Jing B Ping J Chen H Evolution of H9N2 influenza viruses from domestic poultry in Mainland China Virology 2005 340 70 83 10.1016/j.virol.2005.06.025 16026813

[B3] Xu KM Li KS Smith GJ Li JW Tai H Zhang JX Webster RG Peiris JS Chen H Guan Y Evolution and molecular epidemiology of H9N2 influenza A viruses from quail in southern China, 2000 to 2005 J Virol 2007 81 2635 2645 10.1128/JVI.02316-06 17192315 PMC1865985

[B4] Xu KM Smith GJ Bahl J Duan L Tai H Vijaykrishna D Wang J Zhang JX Li KS Fan XH The genesis and evolution of H9N2 influenza viruses in poultry from southern China, 2000 to 2005 J Virol 2007 81 10389 10401 10.1128/JVI.00979-07 17652402 PMC2045440

[B5] Sun Y Pu J Jiang Z Guan T Xia Y Xu Q Liu L Ma B Tian F Brown EG Liu J Genotypic evolution and antigenic drift of H9N2 influenza viruses in China from 1994 to 2008 Vet Microbiol 2010 146 215 225 10.1016/j.vetmic.2010.05.010 20685047

[B6] Zhang P Tang Y Liu X Liu W Zhang X Liu H Peng D Gao S Wu Y Zhang L Novel Genotype H9N2 Influenza Virus Possessing Human H5N1 Internal Genomes Has Been Circulating in Poultry in Eastern China since 1998 J Virol 2009 83 8428 8438 10.1128/JVI.00659-09 19553328 PMC2738149

[B7] Choi YK Ozaki H Webby RJ Webster RG Peiris JS Poon L Butt C Leung YH Guan Y Continuing evolution of H9N2 influenza viruses in Southeastern China J Virol 2004 78 8609 8614 10.1128/JVI.78.16.8609-8614.2004 15280470 PMC479067

[B8] Sun Y Qin K Wang J Pu J Tang Q Hu Y Bi Y Zhao X Yang H Shu Y Liu J High genetic compatibility and increased pathogenicity of reassortants derived from avian H9N2 and pandemic H1N1/2009 influenza viruses Proc Natl Acad Sci USA 2011 108 4164 4169 10.1073/pnas.1019109108 21368167 PMC3054021

[B9] Bi J Deng G Dong J Kong F Li X Xu Q Zhang M Zhao L Qiao J Phylogenetic and molecular characterization of H9N2 influenza isolates from chickens in Northern China from 2007-2009 PLoS One 2010 5 e13063 10.1371/journal.pone.0013063 PMC2947496 20927364

[B10] Butt AM Siddique S Idrees M Tong Y Avian influenza A (H9N2): computational molecular analysis and phylogenetic characterization of viral surface proteins isolated between 1997 and 2009 from the human population Virol J 2010 7 319 10.1186/1743-422X-7-319 21078137 PMC2994543

[B11] Matrosovich MN Krauss S Webster RG H9N2 influenza A viruses from poultry in Asia have human virus-like receptor specificity Virology 2001 281 156 162 10.1006/viro.2000.0799 11277689

[B12] Wan H Sorrell EM Song H Hossain MJ Ramirez-Nieto G Monne I Stevens J Cattoli G Capua I Chen LM Replication and transmission of H9N2 influenza viruses in ferrets: evaluation of pandemic potential PLoS One 2008 3 e2923 10.1371/journal.pone.0002923 18698430 PMC2500216

[B13] Huang Y Hu B Wen X Cao S Gavrilov BK Du Q Khan MI Zhang X Diversified reassortant H9N2 avian influenza viruses in chicken flocks in northern and eastern China Virus Res 2010 151 26 32 10.1016/j.virusres.2010.03.010 20347894

[B14] Ge FF Zhou JP Liu J Wang J Zhang WY Sheng LP Xu F Ju HB Sun QY Liu PH Genetic evolution of H9 subtype influenza viruses from live poultry markets in Shanghai, China J Clin Microbiol 2009 47 3294 3300 10.1128/JCM.00355-09 19656985 PMC2756938

[B15] Guan Y Shortridge KF Krauss S Chin PS Dyrting KC Ellis TM Webster RG Peiris M H9N2 influenza viruses possessing H5N1-like internal genomes continue to circulate in poultry in southeastern China J Virol 2000 74 9372 9380 10.1128/JVI.74.20.9372-9380.2000 11000205 PMC112365

[B16] Iqbal M Yaqub T Reddy K McCauley JW Novel genotypes of H9N2 influenza A viruses isolated from poultry in Pakistan containing NS genes similar to highly pathogenic H7N3 and H5N1 viruses PLoS One 2009 4 e5788 10.1371/journal.pone.0005788 19517011 PMC2690689

[B17] Lipsitch M Moxon ER Virulence and transmissibility of pathogens: what is the relationship? Trends Microbiol 1997 5 31 37 10.1016/S0966-842X(97)81772-6 9025233

[B18] Ping J Dankar SK Forbes NE Keleta L Zhou Y Tyler S Brown EG PB2 and hemagglutinin mutations are major determinants of host range and virulence in mouse-adapted influenza A virus J Virol 2010 84 10606 10618 10.1128/JVI.01187-10 20702632 PMC2950562

[B19] Scotti PD End-point dilution and plaque assay methods for titration of cricket paralysis virus in cultured Drosophila cells J Gen Virol 1977 35 393 396 10.1099/0022-1317-35-2-393 406358

[B20] Liu J Xiao H Lei F Zhu Q Qin K Zhang XW Zhang XL Zhao D Wang G Feng Y Highly pathogenic H5N1 influenza virus infection in migratory birds Science 2005 309 1206 10.1126/science.1115273 16000410

