# Supplementary material for: Novel genetic reassortants in H9N2 influenza A viruses and their diverse pathogenicity to mice
Source: Virol J. 2011 Nov 4;8:505. doi: 10.1186/1743-422X-8-505 (PMC3236014; doi:10.1186/1743-422X-8-505)
Supplement: Additional file 1 — Figure.S1. Unrooted neighbor-joining phylogenetic trees for the HA (34-1573 nt) (A), NA (20-1387 nt) (B), M (26-1007 nt) (C), NS (27-849 nt) (D), PB2 (28-2289 nt) (E), PB1 (25-2242 nt) (F), PA (25-2175 nt) (G), and NP (46-1526 nt) (H) genes of H9N2 influenza A viruses. Trees were generated by the Maximum Composite Likelihood model of Neighbor-Joining algorithm with MEGA 4.1 http://www.megasoftware.net. The reliability of the trees was assessed by bootstrap analysis with 1, 000 replicates and only bootstrap values ≥ 90% were shown. Different lineages were indicated by different colors. The viruses obtained in the present study were marked with red circles, and the representative strains in each lineage were marked with yellow squares. [file 1743-422X-8-505-S1.DOC]

**Fig. S1A (HA)**

**Fig. S1B (NA)**

**Fig. S1C (M)**

**Fig. S1D (NS)**

**Fig. S1E (PB2)**

**Fig. S1F (PB1)**

**Fig. S1G (PA)**

**Fig. S1H (NP)**
