# Supplementary material for: Novel genetic reassortants in H9N2 influenza A viruses and their diverse pathogenicity to mice
Source: Virol J. 2011 Nov 4;8:505. doi: 10.1186/1743-422X-8-505 (PMC3236014; doi:10.1186/1743-422X-8-505)
Supplement: Additional file 2 — Table S1. Comparison of amino acid sequences of HA, NA, and PB2 genes of representative H9N2 viruses from northern China. [file 1743-422X-8-505-S2.DOC]

**Table S1. Comparison of amino acid sequences of HA, NA, and PB2 genes of** representative H9N2 viruses from northern China

| **Virus** | **Potential glycosylation sites ^a^** | | **RBS ^b^**  **(H3 numbering)** | | | | **HA deletion** | **NA deletion** | **Connecting**  **peptides at HA cleavage site** | **Key sites in PB2 (H3 numbering)** | |
| --- | --- | --- | --- | --- | --- | --- | --- | --- | --- | --- | --- |
|  |  |  | **183** | **190** | **226** | **228** |  |  |  | **627** | **701** |
| Ck/BJ/1/94 | —^c^ | — | N | V | Q | G | —^c^ | —^c^ | PARSSR↓G | E | D |
| Ck/SD/WF/98 | -1 | N551T | N | T | Q | G | — | — | PARSSR↓G | E | D |
| Ck/HLJ/u/98 | — | — | H | E | Q | G | — | 63-65 | PAVSSR↓G | E | D |
| Ck/SD/lx929/07 | — | — | N | V | L | G | 412-418 | — | PARSSR↓G | E | D |
| Ck/SD/lx1023/07 | +1 | G284S | N | A | L | G | — | — | PARSSR↓G | E | D |
| Ck/SD/LY-1/08 | — | — | N | V | Q | G | — | 63-65 | PARSSR↓G | E | D |
| Ck/SD/BD/08 | — | — | N | T | Q | G | — | 63-65 | PARSSR↓G | E | D |
| Ck/SD/02/08 | +1 | P315S | N | A | L | G | — | 63-65 | PARSSR↓G | E | D |
| Ck/SD/01/09 | +2 | P315S  S145N | N | V | L | G | — | 63-65 | PARSSR↓G | E | D |
| Ck/SD/02/09 | +1 | P315S | N | A | L | G | — | 63-65 | PARSSR↓G | E | D |
| Ck/SD/KD/09 | +1 | P315S | N | T | L | G | — | 63-65 | PARSSR↓G | E | D |
| Ck/SD/H/09 | +1 | P315S | N | A | L | G | — | 63-65 | PARSSR↓G | E | D |
| Ck/SD/BD/10 | +1 | P315S | N | T | L | G | — | 63-65 | PARSSR↓G | E | D |
| Ck/SD/01/10 | +1 | P315S | N | V | L | G | — | 63-65 | PARSSR↓G | E | D |
| Ck/SD/02/10 | — | — | N | V | L | G | — | 63-65 | PARSSR↓G | E | D |
| Ck/SD/03/10 | +1 | P315S | N | V | F | G | — | 63-65 | PARSSR↓G | E | D |

^a^ The HA potential glycosylation sites of the 15 isolates were compared with the conserved potential sites of most H9N2 viruses, represented by Ck/BJ/1/94. The additional or decreased numbers of potential glycosylation sites were represented by “+ number” or “- number”, respectively. And the mutated amino acids (H9 numbering) for the change of glycosylation sites were also clarified.

^b^ RBS, receptor binding site.

^c^ —, No addition or deletion in the potential glycosylation sites, or no deletion in the HA / NA protein compared with ck/BJ/1/94.
