# Supplementary material for: Novel genetic reassortants in H9N2 influenza A viruses and their diverse pathogenicity to mice
Source: Virol J. 2011 Nov 4;8:505. doi: 10.1186/1743-422X-8-505 (PMC3236014; doi:10.1186/1743-422X-8-505)
Supplement: Additional file 3 — Table S2. The clinical information of representative avian H9N2 influenza viruses isolated from northern China. [file 1743-422X-8-505-S3.DOC]

**Table S2. The clinical information of representative avian H9N2 influenza viruses isolated from northern China**

| **Virus** | **Abbreviation** | **Collection date** | **Clinical symptoms** | **Host** |
| --- | --- | --- | --- | --- |
| A/chicken/Shandong/WF/1998 | Ck/SD/WF/98 | 1998 | Egg reduction | Layer |
| A/chicken/Heilongjiang/u/1998 | Ck/HLJ/u/98 | 1998 | Egg reduction | Layer |
| A/chicken/Shandong/lx929/2007 | Ck/SD/lx929/07 | 29-Sep-2007 | Mild respiratory symptoms | Layer |
| A/chicken/Shandong/lx1023/2007 | Ck/SD/lx1023/07 | 23-Oct-2007 | Mild respiratory symptoms | Layer |
| A/chicken/Shandong/LY-1/2008 | Ck/SD/LY-1/08 | Dec-2008 | Mild respiratory symptoms, secondary infection | Broiler |
| A/chicken/Shandong/BD/2008 | Ck/SD/BD/08 | Dec-2008 | Egg reduction | Broiler |
| A/chicken/Shandong/02/2008 | Ck/SD/02/08 | Dec-2008 | Respiratory symptoms, secondary infection | Broiler |
| A/chicken/Shandong/01/2009 | Ck/SD/01/09 | Feb-2009 | Respiratory symptoms | Broiler |
| A/chicken/Shandong/02/2009 | Ck/SD/02/09 | Sept-2009 | Egg reduction | Layer |
| A/chicken/Shandong/KD/09 | Ck/SD/KD/09 | Jan-2009 | Egg reduction | Broiler |
| A/chicken/Shandong/H/2009 | Ck/SD/H/09 | May-2009 | Respiratory symptoms | Broiler |
| A/chicken/Shandong/BD/2010 | Ck/SD/BD/10 | May-2010 | Egg reduction | Broiler |
| A/chicken/Shandong/01/2010 | Ck/SD/01/10 | Jan-2010 | Depression, loss of weight, tracheal [bleeding](http://www.iciba.com/bleeding/) and embolism | Broiler |
| A/chicken/Shandong/02/2010 | Ck/SD/02/10 | Feb-2010 | Depression, loss of weight, tracheal [bleeding](http://www.iciba.com/bleeding/) and embolism | Broiler |
| A/chicken/Shandong/03/2010 | Ck/SD/03/10 | May-2010 | Difficulty breathing, secondary infection, high mortality | Broiler |
